# Recent Advances in Deep Learning for Protein-Protein Interaction Analysis: A Comprehensive Review

**DOI:** 10.3390/molecules28135169

**Published:** 2023-07-02

**Authors:** Minhyeok Lee

**Affiliations:** School of Electrical and Electronics Engineering, Chung-Ang University, Seoul 06974, Republic of Korea; mlee@cau.ac.kr

**Keywords:** deep learning, protein–protein interactions, computational biology, artificial intelligence, PPI prediction, bioinformatics, machine learning, AI, protein networks

## Abstract

Deep learning, a potent branch of artificial intelligence, is steadily leaving its transformative imprint across multiple disciplines. Within computational biology, it is expediting progress in the understanding of Protein–Protein Interactions (PPIs), key components governing a wide array of biological functionalities. Hence, an in-depth exploration of PPIs is crucial for decoding the intricate biological system dynamics and unveiling potential avenues for therapeutic interventions. As the deployment of deep learning techniques in PPI analysis proliferates at an accelerated pace, there exists an immediate demand for an exhaustive review that encapsulates and critically assesses these novel developments. Addressing this requirement, this review offers a detailed analysis of the literature from 2021 to 2023, highlighting the cutting-edge deep learning methodologies harnessed for PPI analysis. Thus, this review stands as a crucial reference for researchers in the discipline, presenting an overview of the recent studies in the field. This consolidation helps elucidate the dynamic paradigm of PPI analysis, the evolution of deep learning techniques, and their interdependent dynamics. This scrutiny is expected to serve as a vital aid for researchers, both well-established and newcomers, assisting them in maneuvering the rapidly shifting terrain of deep learning applications in PPI analysis.

## 1. Introduction

In the current era, Artificial Intelligence (AI) forms a transformative underpinning of our scientific progress [1,2,3]. Leveraging advancements in generative deep learning architectures, such as Generative Adversarial Networks (GANs) [4,5,6,7,8], Neural Radiance Fields (NeRF) [9,10,11,12,13,14], and models such as the Generative Pre-training Transformer (GPT) [15,16,17,18], we are facing the proposition that creative intuition, once perceived as an exclusive human trait, may potentially be replicated or even surpassed within an algorithmic framework.

Deep learning has demonstrated exceptional prowess in uncovering complex patterns within high-dimensional data, resulting in ground-breaking applications across various domains [19,20,21]. By exploiting multiple layers of non-linear processing units for feature extraction and transformation, deep learning models can learn hierarchical representations from vast and complex datasets, a characteristic that has found utility in computational biology [22,23,24], and in particular, the prediction of Protein–Protein Interactions (PPIs).

PPIs, pivotal elements in cellular processes, play an instrumental role in various biological functions [25,26,27,28]. These interactions enable proteins to form complex, dynamic networks, which in turn govern biological phenomena spanning from signal transduction to enzymatic activity. Understanding these interactions is crucial, not only for deciphering the complex orchestration of biological systems but also for the identification of novel therapeutic targets for disease intervention. PPIs can be classified into several categories, each with unique characteristics and functional implications. This classification includes direct (physical) and indirect (functional) interactions, permanent and transient interactions, as well as homomeric and heteromeric interactions. Each of these types of PPIs has distinct attributes and implications, necessitating a thorough understanding for successful prediction and analysis.

One groundbreaking application of deep learning in protein studies is embodied by AlphaFold [29], a remarkable AI system developed by DeepMind. AlphaFold stands as a prime example of the confluence of computational prowess and biological understanding, demonstrating the transformative power of AI in deciphering complex biological systems.

AlphaFold utilizes a deep-learning-based approach to predict protein structure, a problem of profound significance in biology. The AI model has been meticulously trained on a wealth of data derived from the Protein Data Bank, integrating a vast multitude of known protein structures into its learning framework. The system leverages this training to predict the arrangement of amino acids within a protein, generating a comprehensive three-dimensional model that illuminates the protein’s spatial conformation.

With the emerging developments in deep learning, an increasing number of research endeavors have explored its application to PPIs. Deep learning holds the promise of revolutionizing PPI prediction, ushering in an era of highly accurate, efficient, and insightful computational methodologies. This paper, therefore, provides a comprehensive review of the most recent literature that employs deep learning for PPI analysis, with a particular focus on works published during the period of 2021–2023.

In an era where deep learning technologies are experiencing unprecedented growth and innovation, it is imperative to stay abreast of the most recent developments. This review, therefore, serves as a crucial resource for researchers in the field, encapsulating the state-of-the-art techniques in PPI analysis using deep learning, thereby providing insights into this rapidly evolving domain.

## 2. Literature Review Methods

### 2.1. Study Selection Process

The primary objective of the paper selection process was to ascertain the incorporation of high-quality, related research in the deep learning for PPIs domain. This was accomplished by adopting an algorithmic approach primarily hinged on the scholarly search engine, Web of Science (WOS). The search keywords were meticulously selected, focusing on crucial topics such as “deep learning”, “protein–protein interactions”, and “artificial neural network”. This was done with the intention of identifying pertinent articles for a comprehensive review. The review is strictly confined to papers published in peer-reviewed journals. This restriction was instated on account of two main reasons. First, peer-reviewed journals typically uphold the quality and reliability of the scientific literature by subjecting the papers to an intense review by experts in the field. Second, they are considered trustworthy sources for the publication of scientifically robust and influential research.

Despite acknowledging the presence of preprints and conference papers in this domain, it was decided to concentrate solely on peer-reviewed journal articles. This decision was motivated by the need to enhance the reliability and validity of the review, by ensuring the inclusion of studies that have undergone an intense review process. Moreover, in order to retain the novelty and originality of the review, certain article types like review articles and perspectives were deliberately excluded. The aim was to emphasize the integration of primary research-based studies, aligning with the purpose of the review.

The temporal scope of the review was restricted to articles published during the last three years, from 2021 to 2023. This timeframe was selected to guarantee the relevance and contemporaneity of the review. This allows for a thorough understanding of the most recent developments and trends in deep learning for PPIs. It is important to mention that the data collection for 2023 was conducted up to May, in line with the present timeline, thereby ensuring that the review remains concurrent with the latest advancements in the field. Throughout the data collection process, we gathered information about the number of citations and the publication log for each selected article. These details served as vital factors in appraising the scope, impact, and acceptability of the research within the scientific community.

To provide a structured overview of deep learning for PPIs, the selected papers were categorized based on the objectives of the specific studies. This classification contributes to a comprehensive understanding of the varied methodologies employed in the field, thereby enhancing our understanding of the deep learning landscape for PPIs. Despite many papers aligning with multiple categories, they were assigned to a single category that best represented the main theme of the paper. Table 1 presents a summary of the reviewed papers.

### 2.2. An Analysis of Selected Papers

An examination of the selected papers was undertaken to elucidate the utilization of deep learning methodologies for protein–protein interactions. These deep learning techniques have been enumerated in Table 1 alongside a brief description and their corresponding studies.

*Graph Neural Networks* have been exceedingly utilized for deep learning applications in numerous studies, capitalizing on graph data processing. They have been effectively employed to model PPIs, given the inherent graph-like structure of protein interaction networks. A non-negligible portion of the studies employed *Convolutional Neural Networks*. Capitalizing on their capacity for spatial data processing, CNNs have been utilized for deep learning purposes in PPI research. In a different vein, certain studies have exploited *Representation Learning and Autoencoders* for obtaining representations with deep learning, which has proven instrumental in discerning novel features and protein interaction patterns. The sequential data processing capabilities of *Recurrent Neural Networks, including Long Short-Term Memory networks*, have been harnessed in various studies, which underscores their utility in handling time-series data and capturing temporal dependencies, a feature especially relevant for sequential biological data such as protein sequences. Noteworthy is the application of *Attention Methods and Transformers* that rely on the attention mechanism and position-specific encoding for deep learning. Their ability to model long-range interactions and complex dependencies makes them suitable for tasks such as predicting PPIs. Moreover, *Multi-task and Multi-modal Learning* methods have found their application in a number of studies. These methods can effectively handle multiple tasks or data types simultaneously, thus, they can simultaneously predict multiple types of PPIs or utilize different kinds of biological data. Several studies have adopted *Transfer Learning* approaches, reaping the benefits of pre-trained deep learning models for feature extraction, and thus reducing the requirement for vast quantities of training data.

The category of *Generic/Applications (including Multi-Layer Perceptrons (MLPs)) and Others* encompasses a broad range of models and applications, including some that do not specifically fit into the aforementioned categories or those that use PPIs as inputs for deep learning models. This signifies the breadth and diversity of deep learning applications in the field of PPIs. The landscape of deep learning for PPIs is marked by a diverse array of methodologies, each having its unique capabilities and advantages, which have been adeptly utilized in various studies for unveiling the complex patterns of protein interactions.

### 2.3. Journals of Publications

The journals in which the selected papers were published provide insight into the scientific communities that are actively engaged in deep learning for PPIs. An analysis of the publication outlets for these articles can also shed light on their impact and reach within the scientific community.

Table 2 presents a breakdown of the journals where the selected articles were published. The journal ’Bioinformatics’ featured the highest count with 21 articles, constituting 17.6% of the total publications. This indicates the journal’s significant role in promulgating research on deep learning for PPIs.

The ’Briefings in Bioinformatics’ and ’BMC Bioinformatics’ journals both housed 12 publications, each comprising 10.1% of the total reviewed articles. This underscores their substantial contribution to the dissemination of research in this field.

The ’IEEE-ACM Transactions on Computational Biology and Bioinformatics’ journal, with seven articles, constitutes 5.9% of the total publications. This suggests a substantial interest in this topic within the computational biology and bioinformatics community.

The ’Computational and Structural Biotechnology Journal’ and ’Frontiers in Genetics’, each with four articles, represents 3.4% of the total papers reviewed, indicating their role in the research landscape of deep learning for PPIs.

A host of other journals, each with two publications, embody 1.7% of the total, including prestigious titles like ’Science’ and ’Nature Machine Intelligence’, highlighting the interdisciplinary and cross-field interest in this research area.

### 2.4. Year and Citations of Publications

**Figure 1:**
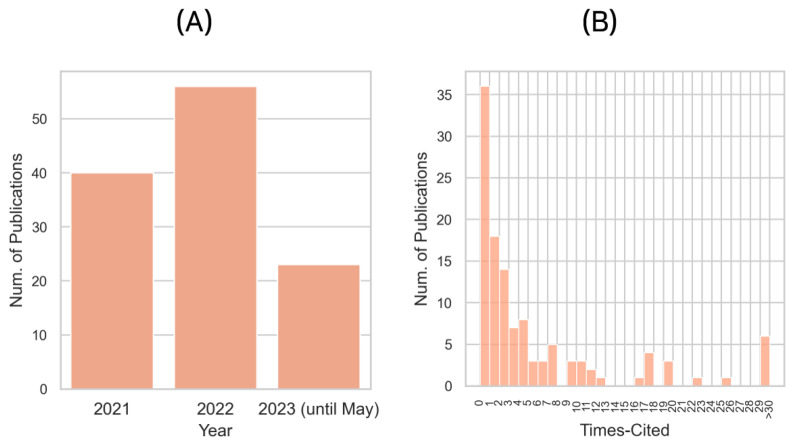
Overview of the Distribution of Publication Years and Citation Frequencies. (**A**) Illustrates the distribution of publication years; (**B**) displays the distribution of citation frequencies.

The dynamics of publications in the domain of deep learning for PPIs can be analyzed in terms of temporal distribution. As illustrated in Figure 1, the number of publications has seen a remarkable increase over the years, reflecting the growing interest in and significance of this research area.

In 2021, a total of 40 studies were published that applied deep learning methods to PPIs. This represents a significant contribution to the field, reflecting a mature state of research interest. The following year, 2022, witnessed a substantial surge in the number of publications, amounting to 56. This represents an approximately 40% increase from the previous year. This rapid growth signals the emerging enthusiasm and considerable advancements in the application of deep learning methods to PPIs.

As for 2023, until May, there have already been 23 papers published. If the current publication rate persists throughout the year, the total number of publications in 2023 is projected to surpass that of the previous years. This trend underlines the continuous evolution of the field, as well as the persistent pursuit for improved methodologies for understanding and leveraging PPIs using deep learning techniques.

An examination of citation distribution offers insights into the reception and influence of publications within the sphere of deep learning for PPIs. Statistical metrics, such as median and mean, can provide a robust summary of the overall citation landscape. The median number of citations for these publications is recorded as 2, while the mean is observed to be slightly higher at 5.3. A notable point is the high number of studies that have not yet been cited, implying that these are relatively recent contributions, or perhaps they have yet to be discovered or appreciated by the wider research community. This lack of citations may also be an artifact of the current data collection process, as data for 2023 is not fully collected and updated by the WoS.

The disparity between the mean and median citation count can be indicative of a skewed distribution, likely due to a small number of highly cited papers. It highlights the breadth of research impact, where a handful of studies may have profoundly influenced the field, while the majority of studies are yet to make a substantial impact. These findings, combined with the awareness that the field is still young and in a constant state of evolution, paint a promising picture for the future of deep learning applications in PPIs. It reinforces the idea that this research area is rich with opportunity and potential for transformative discoveries.

Overall, the increasing trend in the number of publications underscores the vitality of this research domain and implies the potential for future development. This continuous growth reflects the ongoing refinement of deep learning methods applied to PPIs and the recognition of their valuable contributions in biological and computational research.

## 3. Historical Deep Learning Methods for Protein–Protein Interaction Analysis

The emergence and development of historical deep learning methodologies for PPI analysis have significantly facilitated the comprehensive understanding of complex cellular processes. They have been instrumental in enabling thorough investigation and prediction of these interactions. In this section, two representative frameworks (PIPR and DPPI) and their limitations are discussed.

The PIPR framework [149] introduces an innovative approach for PPI prediction centered around amino acid sequences. This method is anchored in a Siamese architecture, leveraging a deep residual recurrent convolutional neural network (RCNN). The integration of recurrent and convolutional layers allows PIPR to accurately capture fundamental local and sequential attributes inherent in protein sequences. To further augment the feature extraction process, PIPR employs an automatic multi-granular feature selection mechanism. This assists PIPR in identifying and giving precedence to the most informative and distinguishing features within the sequences. In addition to this, PIPR amalgamates diverse aspects of PPI data, which includes sequence similarity, evolutionary preservation, and domain-domain interactions, to establish a comprehensive and thorough predictive model. The DPPI model addresses both homodimeric and heterodimeric protein interactions. It can also replicate binding affinities. The creation of the RCNN employed bidirectional gated recurrent units (i.e., bidirectional-GRU), yet GRUs have demonstrated limited learning efficiency and slow convergence [150].

The DPPI method [151] introduces a distinct approach for PPI prediction by harnessing deep learning techniques. The use of deep Siamese-like CNNs, combined with random projection and data augmentation, allows DPPI to deliver accurate sequence-based PPI predictions. This method concentrates on capturing critical aspects of a protein pair’s composition, which includes the amino acid sequence and the co-occurrence of overlapping sequence motifs. DPPI employs PSI-BLAST to generate probabilistic sequencing profiles for each protein to extract pertinent features, offering a holistic description. The convolutional module, made up of multiple layers, identifies sequence patterns within each protein’s profile. Furthermore, DPPI applies random projection to the representations sourced from the convolutional module, projecting them into two unique spaces. The Siamese-based learning architecture captures the reciprocal influence of protein pairings, allowing for generalization in addressing diverse PPI prediction problems without the necessity for predefined features. However, based on 5-fold cross-validation, DPPI’s performance in terms of PPI prediction accuracy on the S.cerevisiae core dataset was found to be inferior to that of PIPR [149].

## 4. Graph Neural Networks for Protein–Protein Interactions

Graph Neural Networks (GNNs) [152,153,154,155] have emerged as a versatile and powerful class of methods in the computational prediction of PPIs. They represent a specific form of deep learning architecture specially designed for dealing with data structured as graphs. Given the complex nature of biomolecular data, such as proteins, which can be naturally represented as graphs, GNNs provide a unique opportunity to capture intricate patterns and relationships within these datasets.

In essence, a graph can be seen as a collection of nodes and edges, where nodes represent entities (e.g., proteins), and edges denote relationships or interactions (e.g., PPIs). GNNs take advantage of this structured data format by applying various forms of convolutions directly on the graph, enabling them to learn from both local node features and the broader network topology. This ability is particularly useful in the study of PPIs, where the biological significance of an interaction often depends not only on the properties of the interacting proteins but also on their position and role within the larger protein network.

The unique capacity of GNNs to exploit the underlying structure of graph data is achieved through several key mechanisms. Firstly, GNNs use neighborhood aggregation or message-passing frameworks, wherein each node in the graph gathers information from its local neighbors to update its state. This allows GNNs to incorporate local context into node representations, thereby capturing the immediate interaction dynamics in PPIs. Secondly, through multiple rounds of these aggregations, GNNs can learn increasingly abstract representations of nodes, thereby modeling higher-order interaction effects and uncovering complex interaction patterns.

Various types of GNNs have been employed in the study of PPIs, with each offering unique advantages. Graph Convolutional Networks (GCNs) [156,157,158], for instance, are particularly adept at learning from homophily in networks, wherein nodes that are connected or nearby in the graph have similar features. Graph Attention Networks (GATs) [159,160,161] add another level of sophistication by introducing attention mechanisms that allow different weights to be assigned to different neighbors during the aggregation process. These and other variants of GNNs provide a flexible and robust toolset for tackling the challenging task of PPI prediction.

Research leveraging GNNs for PPI prediction spans a wide range of applications, from identifying specific interaction sites on proteins, predicting the existence of interactions between protein pairs, to classifying proteins based on their interaction profiles. These studies typically involve formulating the PPI problem as a graph-based learning task, such as node classification, link prediction, or graph classification, and employing suitable GNN architectures to solve it.

Recent studies have witnessed a prominent trend in utilizing GNNs for PPI predictions. These studies have explored various models and techniques, aiming to enhance the accuracy and efficiency of PPI prediction tasks. Notably, researchers have focused on leveraging GNNs, such as augmented GATs and GCNs, to capture structural invariance, learn graph representations, and improve prediction performance. Additionally, the integration of multimodal data sources, biological features, and prior knowledge has emerged as a significant aspect of recent research efforts. These studies have demonstrated remarkable advancements in predicting PPIs and utilizing PPI information for various predictive tasks, reinforcing the critical role of deep learning methods, particularly GNNs and GCNs, in advancing our understanding of PPIs and their implications in biological systems. Continued research and methodological advancements are expected to drive further progress in this field. The summary of recent studies can be observed in Table 3.

### 4.1. Pairwise PPI Prediction

Albu et al. [30] presented MM-StackEns, a deep multimodal stacked generalization approach for predicting PPIs, employing a Siamese neural network and graph attention networks, with superior performance on Yeast and Human datasets. Similarly, Jha et al. [36] used Graph Convolutional Network (GCN) and Graph Attention Network (GAT) for PPI prediction, yielding superior results on Human and S. cerevisiae datasets.

### 4.2. PPI Network Prediction

Baranwal et al. [32] offered Struct2Graph, a graph attention network for structure-based PPI predictions, potentially identifying residues contributing to protein–protein complex formation. Gao et al. [34] designed the Substructure Assembling Graph Attention Network (SA-GAT) for graph classification tasks, including potential applications in PPI networks. Zaki et al. [50] proposed a method for detecting protein complexes in PPI data using GCNs, formulating protein complex detection as a node classification problem and implementing the Neural Overlapping Community Detection (NOCD) model.

### 4.3. PPI Site Prediction

Quadrini et al. [40] used Graph Convolutional Networks for PPI site prediction, exploring a novel abstraction of protein structure termed as hierarchical representations. Mahbub and Bayzid [39] introduced EGRET, an edge aggregated graph attention network for PPI site prediction, reporting significant improvements in performance. Yuan et al. [49] proposed GraphPPIS, a deep graph-based framework for PPI site prediction that delivered significantly improved performance over other methods.

### 4.4. Docking

Williams et al. [48] developed DockNet, a high-throughput protein–protein interface contact prediction model utilizing a Siamese graph-based neural network. Reau et al. [41] developed DeepRank-GNN, a graph neural network framework that converts protein–protein interfaces into graphs to learn interaction patterns.

### 4.5. Auxiliary PPI Prediction Tasks

Azadifar and Ahmadi [31] introduced a semi-supervised learning method based on GCNs for prioritizing candidate disease genes. Dai et al. [33] formulated PIKE-R2P, a graph neural network method incorporating PPIs for predicting protein abundance from scRNA-seq data. Hinnerichs and Hoehndorf [35] developed DTI-Voodoo, a method combining molecular features and PPI networks to predict drug-target interactions. Kim et al. [37] proposed DrugGCN for drug response prediction using gene expression data. Wang et al. [46] developed SIPGCN, a GCN-based model for predicting self-interacting proteins (SIPs) from sequence information.

The range and depth of these studies underscore the crucial role deep learning methods, particularly GNNs and GCNs, continue to play in advancing PPI predictions. With ongoing research and methodological enhancements, the future promises continued progress in understanding and predicting PPIs and their influence on biological systems.

## 5. Convolutional Neural Networks for Protein–Protein Interactions

Convolutional Neural Networks (CNNs) [162,163,164] represent another major deep learning architecture that has found substantial application in the prediction of PPIs. Inspired by the organization of the animal visual cortex, CNNs are specialized kinds of neural networks for processing data with a grid-like topology, such as an image, which can also be extended to handle 1D sequence data, like protein sequences, or 3D data, like protein structures.

A CNN typically consists of multiple layers, which may include convolutional layers, pooling layers, and fully connected layers. The distinctive feature of CNNs is the convolutional layer that performs a convolution operation. In the context of a 1D sequence such as a protein sequence, a convolution involves a filter (or kernel) moving across the input sequence and performing an element-wise multiplication and sum operation, thereby capturing local dependencies within the sequence. In the case of 2D data like images or 3D data like protein structures, similar operations are performed but in two or three dimensions, respectively.

This local receptive field, embodied in the convolution operation, allows the model to automatically and adaptively learn spatial hierarchies of features. For instance, lower layers of the network might learn to recognize simple patterns such as certain sequence motifs in a protein sequence, while higher layers could learn to recognize more complex patterns based on the lower-level features, analogous to recognizing complex shapes or objects from simple edges in image data.

Pooling layers within a CNN perform a down-sampling operation along the spatial dimensions, which helps to make the representation invariant to small translations and reduce the computational complexity. The fully connected layers typically come towards the end of the network and can be seen as a traditional multi-layer perceptron that uses the high-level features extracted by the preceding convolutional and pooling layers to perform classification or regression.

In PPIs, CNNs are often employed to learn from protein sequence or structure data, where they can effectively capture local dependencies and hierarchies of biological features. For instance, studies in this category might involve predicting whether a given pair of proteins interacts based on their sequence or structural features, or identifying the specific sites of interaction on a given protein.

Additionally, CNNs have been combined with other types of networks, such as RNNs or attention networks, to better model complex dependencies in the data. These hybrid models allow researchers to leverage the strengths of multiple architectures to improve PPI prediction performance.

Recent studies have showcased notable trends in the application of CNNs for PPI analysis. These studies have explored diverse models and approaches, aiming to enhance the accuracy and effectiveness of PPI prediction tasks. Researchers have developed deep residual neural networks, ensemble residual CNNs, and Siamese-ensemble models, among others, to leverage sequence-driven features, improve prediction performance, and circumvent local optima. Additionally, the application of CNNs in protein docking, binding site prediction, and human-virus PPI analysis has demonstrated significant advancements. The integration of deep learning frameworks, such as recurrent CNNs and three-track neural networks, has proven valuable in predicting protein interactions, phosphorylation sites, and protein–peptide binding sites. Moreover, advancements in protein sequence encoding formats and graph-regularized CNNs have contributed to the coherence and biological interpretation of gene clusters in spatial gene expression analysis. The range and depth of these studies highlight the importance of CNNs in advancing our understanding and prediction of PPIs, emphasizing their potential for future research endeavors. Table 4 provides an overview of the latest research findings.

### 5.1. Pairwise PPI Prediction

Chen et al. [53] designed the Double-Channel-Siamese-Ensemble (DCSE) model, a sequence-based computational approach, for pairwise PPI prediction, with superior performance. Additionally, Gao et al. [54] developed EResCNN, a predictor for PPIs based on an ensemble residual convolutional neural network, outperforming existing models in PPI prediction on various datasets. Hu et al. [56] developed DeepTrio, a PPI prediction tool using mask multiple parallel convolutional neural networks, outperforming several state-of-the-art methods.

### 5.2. PPI Network Prediction

Yuan et al. [65] introduced a deep-learning-based approach for constructing complete PPI networks. By combining a semi-supervised SVM classifier and a CNN, they facilitated protein complex detection with superior performance on benchmark datasets.

### 5.3. PPI Site Prediction

Hu et al. [57] presented D-PPIsite, a deep residual neural network for PPI site prediction. It achieved superior performance with an average accuracy of 80.2% and precision of 36.9% when tested on five independent datasets.

### 5.4. Docking

Guo et al. [55] developed TRScore, a 3D RepVGG-based method for ranking protein docking models. This method was designed to improve the accuracy of traditional scoring functions for recognizing near-native conformations. Mallet et al. [59] introduced InDeep, a 3D fully convolutional neural network tool for predicting functional binding sites within proteins. When compared with state-of-the-art ligandable binding site predictors, InDeep exhibited superior performance.

### 5.5. Auxiliary PPI Prediction Tasks

Kozlovskii and Popov [58] developed BiteNet(P)(p), a 3D CNN method for protein–peptide binding site detection. The method is ideal for large-scale analysis of protein–peptide binding sites. Tsukiyama and Kurata [61] proposed Cross-attention PHV, a cross-attention-based neural network for predicting human-virus PPIs. This model outperformed existing models on a benchmark dataset and accurately predicted PPIs for unknown viruses. Song et al. [60] proposed a method for clustering spatially resolved gene expression data using a graph-regularized convolutional neural network. This method leverages the graph of a PPI network, improving the coherence of spatial patterns and providing biological interpretation of the gene clusters in the spatial context.

Wang et al. [62] proposed an enhancement to a 2D CNN for PPI tasks using the Sequence-Statistics-Content (SSC) protein sequence encoding format. Their method enriched unique sequence features to improve the performance of the deep learning model. Xu et al. [63] introduced OR-RCNN, a deep learning framework for PPI prediction based on ordinal regression and recurrent convolutional neural networks. This method outperformed other PPI prediction models when tested on S. cerevisiae and Homo sapiens datasets. Yang et al. [64] developed PhosIDN, an integrated deep neural network for improving the prediction of protein phosphorylation sites. By integrating sequence and PPI information, this model achieved superior performance over existing phosphorylation site prediction methods.

## 6. Representation Learning and Autoencoder for Protein–Protein Interactions

A core challenge in PPIs and related biological properties using deep learning approaches is the representation of the protein sequences or structures. Representation learning [165,166,167], also known as feature learning, is a set of methods that allows a machine or a model to automatically discover the representations needed to classify or predict outcomes from the raw data. This method has proven its effectiveness in various domains, including protein science, by providing an efficient way to transform raw biological data into a format that is suitable for analysis.

In the context of protein studies, representation learning methods have been used to transform protein sequence and structure information into meaningful features that capture the biological properties of the proteins. These methods can range from simple techniques such as one-hot encoding or count-based representations, to more sophisticated methods based on word embeddings like Word2Vec [168,169,170], or even advanced techniques that take into account the sequential nature of proteins, such as RNN embeddings.

Representation learning plays a significant role in PPI analysis by efficiently encoding and representing protein sequences or structures. This involves transforming raw biological data into an informative, reduced-dimensional format that can facilitate further computational analysis and predictive modeling. In the context of PPI studies, this encompasses the development of methodologies and models that convert protein sequences or structural information into meaningful features that capture the essential biological properties of proteins.

The autoencoder [171,172,173], a particular type of artificial neural network, is a powerful tool for representation learning. An autoencoder is designed to learn an efficient encoding and decoding scheme for a set of data, typically aiming to learn a compact representation that preserves as much information about the original data as possible. An autoencoder consists of two parts: the encoder, which maps the input data to a lower-dimensional representation, and the decoder, which reconstructs the original data from this lower-dimensional representation.

By training an autoencoder to minimize the difference between the original and the reconstructed data (known as reconstruction error), we can use the learned lower-dimensional representation as a new feature set for our data. This approach has been particularly useful for PPI prediction, where the complexity and high-dimensionality of protein data often make it difficult to devise hand-crafted features.

Autoencoders can take on various forms depending on the specific use case. For example, denoising autoencoders [174] are trained to reconstruct the original data from a corrupted version of it, making them robust to noise in the input data. Variational autoencoders [171], on the other hand, are a type of generative model that adds a probabilistic spin to autoencoders, allowing them to generate new data that resemble the training data.

A wide range of studies involving PPIs fall within the purview of representation learning and autoencoders. This includes work that uses autoencoders or other representation learning methods to transform protein sequence or structural data into a format suitable for PPI prediction, studies that use these methods to predict specific properties related to PPIs, like interaction sites or interaction types, and those that integrate these methods with other machine learning or deep learning techniques to improve PPI prediction performance.

Recent studies have highlighted the significant role of autoencoders and representation learning in PPI analysis. Researchers have developed innovative frameworks and models that leverage autoencoders to encode protein structures and primary sequences, leading to enhanced computational efficiency and low complexity. Additionally, the integration of graph autoencoders and deep sequence features has demonstrated superior performance in predicting abnormal phenotype-human protein associations. Autoencoders have also been applied in ensemble models for PPI prediction, utilizing separate autoencoders for positive and negative interactions. Representation learning techniques, including hashing methods, have emerged as effective approaches for reducing time complexity in predicting PPI relationships. Deep learning models directly utilizing protein sequences have proven highly accurate, even with limited training data, providing valuable insights into protein characterization. Researchers have also explored interdisciplinary applications, such as viral-host PPI prediction and SARS-CoV2-human host protein interaction analysis, where deep learning methodologies have showcased remarkable advancements. Moreover, the incorporation of GO terms and attention mechanisms has led to the development of models that capture deep semantic relations and outperform traditional semantic similarity measures in PPI prediction. These recent studies collectively underscore the importance of autoencoders and representation learning techniques in advancing our understanding and prediction of PPIs. Recent studies are summarized in Table 5.

### 6.1. Pairwise PPI Prediction

Several models have been proposed to predict pairwise PPIs. Ieremie et al. [69] proposed TransformerGO, which predicts PPIs by modeling the attention between sets of Gene Ontology (GO) terms. Similarly, Jha et al. [70] utilized a stacked auto-encoder for PPI prediction, a deep learning method that accepts a 92-length feature vector derived from protein sequences. Also, Asim et al. [66] introduced LGCA-VHPPI, a deep forest model for viral-host PPI prediction. Moreover, Sledzieski et al. [76] presented D-SCRIPT, a deep-learning model predicting PPIs using only their sequences.

### 6.2. PPI Network Prediction

Several works have focused on the prediction of PPI networks. Hasibi and Michoel [68] demonstrated an end-to-end Graph Feature Auto-Encoder, utilizing the structure of gene networks for prediction of node features. In a similar vein, Jiang et al. [71] proposed DHL-PPI, a deep hash learning model, to predict all-against-all PPI relationships in a database of proteins. In the context of disease, Ray et al. [75] presented a deep learning methodology for predicting high-confidence interactions between SARS-CoV2 and human host proteins.

### 6.3. PPI Site Prediction

Predicting the sites of protein–protein interactions has also been a subject of focus. Wang et al. [78] introduced DeepPPISP-XGB, a method integrating deep learning and XGBoost for the prediction of PPI sites. In another study, Orasch et al. [74] presented a new deep learning architecture based on graph representation learning for predicting interaction sites and interactions of proteins.

### 6.4. Auxiliary PPI Prediction Tasks

Several studies have applied representation learning and autoencoders for auxiliary PPI prediction tasks. Liu et al. [72] designed GraphPheno, a semi-supervised method based on graph autoencoders, for predicting relationships between human proteins and abnormal phenotypes. Nourani et al. [73] presented TripletProt, a deep representation learning approach for proteins, based on Siamese neural networks. Additionally, Yue et al. [79] proposed a deep learning framework integrating features from the PPI network, subcellular localization, and gene expression profiles to identify essential proteins.

Czibula et al. [67] introduced AutoPPI, an ensemble of autoencoders designed for PPI prediction. AutoPPI utilized two autoencoders for positive and negative interactions. Also, Soleymani et al. [77] proposed ProtInteract, a deep learning framework for predicting PPIs, providing low computational complexity and fast response. Both AutoPPI and ProtInteract can be considered general tools applicable to several auxiliary PPI prediction tasks.

## 7. Recurrent Neural Networks for Protein–Protein Interactions

Recurrent Neural Networks (RNNs) are a class of artificial neural networks designed to recognize patterns in sequences of data, such as text, speech, or, in this case, protein sequences [175,176,177]. They offer a powerful tool for processing sequential data due to their inherent ability to “remember” previous inputs in the sequence using hidden states. This memory feature makes RNNs uniquely suitable for tasks where the order of elements is crucial, such as in the prediction of PPIs from protein sequences.

An RNN contains a layer of hidden units, whose activations are calculated based on the current input and the previous hidden state. This recurrent connection allows information to be passed along from one step in the sequence to the next, creating an internal state of the network that allows it to exhibit dynamic temporal behavior.

One of the major variants of RNNs, particularly effective for PPI prediction, is the Long Short-Term Memory (LSTM) network [175]. LSTMs were introduced to combat the “vanishing gradients” problem encountered when training traditional RNNs. They do this by introducing a set of gating mechanisms: the input gate, forget gate, and output gate. These gates, together with a cell state, allow the LSTM to regulate the flow of information through the network.

The cell state acts as a kind of conveyor belt, allowing important information to be carried forward with minimal modification, while the input, forget, and output gates control the extent to which new information is added, old information is removed, and the current state is revealed, respectively. This mechanism allows LSTMs to learn long-term dependencies, making them particularly effective when dealing with protein sequences, which can be quite long and exhibit complex dependencies.

The utility of RNNs, and LSTMs in particular, for the prediction of PPIs is related to the sequential and interdependent nature of protein sequences. The prediction of whether two proteins interact is often dependent not just on the individual amino acids in each protein, but also on the order of these amino acids, and the broader context they are in.

Given this inherent suitability, many studies in PPI prediction use RNNs as a fundamental part of their methodology. This might involve using RNNs to learn a representation of protein sequences, which is then used as input to a prediction algorithm, or integrating RNNs with other machine learning techniques to create hybrid models that combine the strengths of different approaches.

Recent studies have demonstrated notable trends in the utilization of RNNs for PPI analysis. Researchers have developed innovative strategies, such as bidirectional LSTM models, to generate relevant protein sequences and incorporate complex network analysis. Ensembles of deep learning models, including LSTM-based approaches, have showcased superior performance in PPI site prediction by integrating diverse features and auxiliary information. Furthermore, the application of regularization techniques during training has proven effective in improving the accuracy of PPI prediction models. RNNs have also been instrumental in bridging the gap between PPI research and the understanding of complex interactions, such as plant-pathogen interactions and virus–host interactions. Machine learning models incorporating frustration indices, structural features, and word2vec analysis of amino acid sequences have demonstrated promising results in PPI prediction. Deep learning methods have been successfully applied to predict protein interactions related to SARS-CoV-2 and to identify essential proteins. The incorporation of novel features, ensemble models, and network embedding techniques has further improved the accuracy and performance of RNN-based PPI prediction models. Additionally, deep learning approaches have been leveraged for PPI network alignment and sequence-based protein–protein binding predictions, yielding remarkable results and outperforming traditional machine learning methods. These recent studies collectively highlight the significance of RNNs in advancing our understanding and prediction of PPIs, paving the way for further research and innovation in the field. Table 6 presents a condensed version of recent studies.

### 7.1. Pairwise PPI Prediction

Several models have been proposed to predict pairwise PPIs using RNNs. Alakus and Turkoglu [80] proposed a method for predicting protein interactions in SARS-CoV-2 using a protein mapping method inspired by the AVL tree and bidirectional RNNs. Zhang et al. [89] introduced protein2vec, an LSTM-based approach for predicting protein–protein interactions, which outperformed traditional semantic similarity methods. Tsukiyama et al. [87] presented LSTM-PHV, an LSTM model with word2vec for predicting human-virus PPIs.

### 7.2. PPI Site Prediction

Aybey and Gumus [81] proposed SENSDeep, an ensemble deep learning method that integrates different deep learning models including RNNs for predicting PPI sites (PPISs). SENSDeep demonstrated superior performance in various metrics. In a similar vein, Li et al. [83] proposed DELPHI, an ensemble model combining a CNN and a RNN component for PPI-binding sites prediction.

### 7.3. PPI Network Prediction

For PPI network prediction, Mahdipour et al. [84] introduced RENA, an innovative method for PPI network alignment based on recurrent neural networks. Ortiz-Vilchis et al. [85] employed a bidirectional LSTM model for generating relevant protein sequences with partial knowledge of interactions, demonstrating an ability to retain a significant portion of proteins in the original sequence.

### 7.4. Auxiliary PPI Prediction Tasks

Several works have utilized RNNs for auxiliary PPI prediction tasks. Zeng et al. [88] introduced a deep learning framework for identifying essential proteins, using bidirectional LSTMs to capture non-local relationships in gene expression data. Similarly, Szymborski and Emad [86] introduced RAPPPID, an AWD-LSTM twin network designed for predicting protein–protein interactions, which outperformed other methods on stringent interaction datasets composed of unseen proteins. Zhou et al. [90] implemented an LSTM model for PPI prediction based on frustration, a statistical potential.

## 8. Attention Mechanism and Transformer for Protein–Protein Interactions

The attention mechanism and transformer networks represent breakthroughs in the field of deep learning and have proven to be highly effective for a variety of applications [15,16,17,18], including the prediction of PPIs. At the core of these methodologies is the capability to handle sequence data, recognize patterns, and assign varying importance to different parts of the input data.

The attention mechanism was introduced to improve the performance of recurrent neural network architectures, particularly in tasks dealing with sequences of data. The central idea behind the attention mechanism is to allow the model to focus on different parts of the input sequence when producing an output. This is done by assigning weights, or “attention scores,” to different elements in the sequence, which determine the amount of attention each element should receive. The attention scores are computed dynamically and depend on the context within which the data is processed. This concept allows the model to ’focus’ on relevant parts of the input for each step in the output sequence, thereby improving its ability to handle long sequences and complex dependencies.

The transformer network, on the other hand, represents a new class of model architectures that exclusively use attention mechanisms, eliminating the need for recurrence altogether. Proposed by Vaswani et al. [178], the transformer model is composed of a stack of identical layers, each of which has two sub-layers: a multi-head self-attention mechanism, and a simple, position-wise fully connected feed-forward network.

In the multi-head attention mechanism, the model computes attention scores multiple times with different learned linear projections of the input. This allows the model to focus on different types of information in different parts of the input sequence. Meanwhile, the position-wise feed-forward networks are applied identically to each position, allowing the model to learn complex patterns within the sequence.

For the prediction of PPIs, these methodologies provide significant advantages. Due to their ability to capture dependencies regardless of their distance in the sequence, attention mechanisms and transformers can efficiently process protein sequences, which are inherently sequential and can exhibit complex, long-range dependencies. This makes them well-suited to tasks that involve recognizing patterns in protein sequences to predict whether and how proteins interact.

Given their effectiveness and versatility, attention mechanisms and transformer models have been used in a variety of ways in PPI prediction. Some studies employ these methods to learn robust representations of protein sequences, while others incorporate them into more complex models designed to leverage different types of biological information for PPI prediction.

Recent studies have showcased the growing popularity of attention mechanisms and Transformer models in the field of PPI prediction. Researchers have explored innovative approaches that integrate attention mechanisms into deep learning architectures to improve the accuracy and performance of PPI prediction models. These attention-based models have demonstrated remarkable results across various datasets and tasks. The integration of attention mechanisms with LSTM, convolutional, and self-attention layers has yielded powerful hybrid models for PPI prediction. Moreover, the utilization of Transformer neural network architectures, originally designed for natural language processing, has shown great potential in pre-training sequence representations and fine-tuning them for specific PPI-related tasks. The effectiveness of attention networks and Transformer models is evident in their superior performance compared to existing computational methods for PPI site prediction, protein interaction prediction, bio-entity relation extraction, and protein interaction network reconstruction. These recent studies highlight the significance of attention mechanisms and Transformer models in advancing our understanding and prediction of PPIs, paving the way for further research and development in the field. The findings of recent studies are outlined in Table 7.

### 8.1. Pairwise PPI Prediction

Several studies have leveraged the power of attention mechanisms and transformers for pairwise PPI prediction. Asim et al. [91] proposed ADH-PPI, a deep hybrid model that uses a combination of long short-term memory, convolutional, and self-attention layers. Li et al. [94] introduced SDNN-PPI, a method that employs self-attention to enhance deep neural network feature extraction from protein sequences. Nambiar et al. [95] presented a Transformer neural network for pre-training task-agnostic sequence representations, which was fine-tuned for protein interaction prediction tasks.

### 8.2. PPI Site Prediction

In the domain of PPI site prediction, Tang et al. [96] proposed HANPPIS, a novel hierarchical attention network structure that integrates six effective features of protein sequence into its predictive model, demonstrating superior performance compared to other computational methods.

### 8.3. PPI Network Prediction

For PPI network prediction, Zhu et al. [100] introduced the Structural Gated Attention Deep (SGAD) model, a deep-learning-based framework that leverages multiple protein sequence descriptors, topological features, and information flow of the PPI network.

### 8.4. Auxiliary PPI Prediction Tasks

Several models have been developed for auxiliary PPI prediction tasks. Li et al. [93] utilized a Transformer for embedding words of a sentence into distributed representations for PPI relationship extraction. Zhang and Xu [99] introduced a multiple kernel ensemble attention method for graph learning applied to PPIs, which automatically learns the optimal kernel function from a set of predefined candidate kernels. Warikoo et al. [97] presented LBERT, a lexically aware transformer-based bidirectional encoder representation model for bio-entity relation extraction (BRE). Wu et al. [98] proposed CFAGO, a protein function prediction method that integrates single-species PPI networks and protein biological attributes via a multi-head attention mechanism.

### 8.5. Protein Docking

Baek et al. [92] utilized a three-track neural network that integrates information at different dimensional levels for protein structure and interaction prediction, showing nearly comparable performance to DeepMind’s system in the 14th Critical Assessment of Structure Prediction (CASP14) conference.

## 9. Multi-task or Multi-modal Deep Learning Models for Protein–Protein Interactions

The utilization of multi-task and multi-modal deep learning models [179,180] has been increasingly recognized as an efficient approach to deal with the complexity and heterogeneity of PPI prediction problems. These models are designed to leverage multiple related tasks or multiple sources of information to improve predictive performance, offering a promising direction for the exploration and prediction of PPIs.

Multi-task learning models are designed to improve learning efficiency and predictive performance by learning multiple related tasks concurrently [179]. The fundamental concept behind multi-task learning is the sharing of representations among tasks, which can improve the generalization performance by leveraging the commonalities and differences across tasks. In a standard multi-task learning framework, each task has its own specific layers (task-specific layers), while some layers (shared layers) are shared among all tasks. During training, each task’s loss function is typically optimized, and the overall objective is a weighted sum of these individual loss functions. The shared layers learn a representation that captures the common features among tasks, while the task-specific layers learn the unique features for each task.

Multi-modal deep learning models [180], on the other hand, aim to integrate information from multiple sources or modes. The basic principle of multi-modal learning is to construct a joint representation that leverages the complementarity and correlation among different modalities to improve prediction performance. In a standard multi-modal learning framework, the model first learns a representation for each modality using modality-specific layers and then integrates these representations using shared layers. The modalities can be different types of data (e.g., sequence data, structure data), each of which provides a unique perspective on the problem.

In the context of PPI prediction, these methodologies offer several advantages. Multi-task learning models can learn from multiple related tasks (e.g., predicting different types of protein interactions), thereby leveraging the shared information among tasks to improve prediction performance. Similarly, multi-modal models can integrate information from multiple sources (e.g., sequence data, structural data, functional data), thereby leveraging the complementarity among different types of data to obtain a more comprehensive understanding of the protein interaction mechanisms.

Given their potential for dealing with complex and heterogeneous PPI prediction problems, multi-task and multi-modal deep learning models have found broad applications in the PPI field. They have been used to leverage multiple related tasks or multiple sources of information, improving prediction performance and providing a more comprehensive understanding of the protein interaction mechanisms.

Recent studies have focused on the development of multi-task or multi-modal deep learning models to enhance the prediction of PPIs. These models aim to leverage multiple sources of information, such as protein sequences, structural annotations, gene features, multiomics data, and GO information, to improve the accuracy and robustness of PPI predictions. By incorporating various tasks or modalities into the learning process, these models have demonstrated superior performance compared to single-task methods. Additionally, efforts have been made to enhance the interpretability of deep learning models by incorporating explainable features or methodologies. These advancements in multi-task and multi-modal deep learning approaches have opened up new possibilities for predicting PPIs and expanding our understanding of complex biological interactions in diverse areas, including disease research and infectious disease studies. Table 8 outlines the main points from recent research.

### 9.1. Pairwise PPI Prediction

A range of models have been proposed to predict pairwise PPIs. For instance, Capel et al. [101] proposed a multi-task learning strategy to predict residues in PPI interfaces from protein sequences. Similarly, Li et al. [102] developed EP-EDL, an ensemble deep learning model, to predict human essential proteins using protein sequence information. Thi Ngan Dong et al. [107] employed a multitask transfer learning approach for predicting PPIs between viruses and human cells, showing the effectiveness of this method across multiple PPI prediction tasks.

### 9.2. PPI Network Prediction

Several models have been developed to predict PPI networks. Peng et al. [105] introduced MTGCN, a multi-task learning method based on the Graph Convolutional Network, to identify cancer driver genes using gene features from the PPI network. Schulte-Sasse et al. [106] developed EMOGI, which utilizes graph convolutional networks to integrate multiomics pan-cancer data with PPI networks for cancer gene prediction. Finally, Pan et al. [104] proposed DWPPI, a network embedding-based approach that integrates deep neural networks for PPI prediction in plants, demonstrating superior performance across multiple datasets.

### 9.3. PPI Site Prediction

In the PPI site prediction, Capel et al. [101] have demonstrated a promising approach, utilizing a multi-task learning strategy to predict residues in PPI interfaces from protein sequences, outperforming single-task methods significantly.

### 9.4. Auxiliary PPI Prediction Tasks

A variety of models have been proposed for auxiliary PPI prediction tasks. Linder et al. [103] introduced scrambler networks, a feature attribution method designed for discrete sequence inputs, to improve the interpretability of neural networks for biological sequences. These networks have been used for interpreting effects of genetic variants, cis-regulatory elements interactions, and PPI binding specificity. Lastly, Zheng et al. [108] developed DeepAraPPI, an integrative deep learning framework for predicting PPIs in Arabidopsis thaliana, demonstrating excellent performance and promising cross-species predictive ability.

## 10. Transfer Learning for Protein–Protein Interactions

Transfer learning [181,182,183], a crucial paradigm in machine learning, has drawn increasing attention in the field of PPIs prediction due to its effectiveness in dealing with limited labeled data scenarios. The primary objective of transfer learning is to leverage the knowledge gained from one or more source tasks to improve the learning performance on a target task. The principle behind this approach is the recognition that the learned knowledge in one task can be reused in another related task, therefore facilitating efficient learning.

In the context of a typical transfer learning framework, the initial training phase occurs on a source task or tasks, from which a model learns generic representations. Once the model is trained on the source task, the learned knowledge, typically in the form of model parameters or learned representations, is then transferred to the target task. This transfer step can be realized in different ways. One common approach is to use the trained model on the source task as a pre-trained model for the target task, either by fine-tuning the entire model or by freezing some layers (typically the lower layers) and training only the remaining ones (typically the higher layers).

There are several key reasons why transfer learning can be advantageous for PPI prediction. One fundamental reason is that it enables the use of large amounts of labeled data available for some tasks (source tasks) to assist the learning process in other tasks (target tasks) that have limited labeled data. This is particularly useful in the field of bioinformatics where obtaining labeled data can be expensive and time-consuming. Additionally, transfer learning can help to mitigate the risk of overfitting on small datasets by introducing prior knowledge into the model.

Transfer learning models can be categorized into different types based on the nature of the source and target tasks and the relationship between them. Examples of categories include inductive transfer learning, transductive transfer learning, and unsupervised transfer learning. In the field of PPIs, the use of transfer learning is typically seen in tasks where there is a need to leverage knowledge from well-studied organisms or proteins to less-studied ones, or from one type of protein interaction to another.

A host of studies [109,110,111,112,113] have demonstrated the potential of these methodologies to enhance our understanding of PPI mechanisms and to develop predictive models with superior accuracy. A summary of recent research can be seen in Table 9.

Among these, Chen et al. [109] put forward TNNM, a transfer neural-network-based model specifically designed for the prediction of essential proteins. The researchers achieved this by extracting raw features from multiple biological data sources and demonstrating enhanced prediction performance compared to existing models. This approach represents a significant contribution to the field, particularly considering the critical role that essential proteins play in sustaining cellular life. Similarly, Si and Yan [111] made strides in the prediction of inter-protein contacts, introducing a deep learning method known as DRN-1D2D_Inter. The model leveraged pretrained protein language models, generating enriched input features and achieving superior performance compared to existing state-of-the-art methods. Remarkably, the model maintained its high performance even when predictions were made purely from sequences. Further, the researchers demonstrated the practicality of their model by applying predicted contacts as constraints for protein–protein docking, significantly improving protein complex structure prediction.

Along the same lines, Zhang et al. [113] developed a deep learning framework named HDIContact. The model, designed to predict inter-protein residue contacts using sequence information, utilized transfer learning to generate a two-dimensional Multiple Sequence Alignment (MSA) embedding. The researchers tested HDIContact on an Escherichia coli dataset, where it outperformed other state-of-the-art methods. This advancement shows promising implications for understanding PPI mechanisms.

Turning attention towards the identification and characterization of protein structural sites, Derry and Altman [110] proposed COLLAPSE, a deep learning framework that operates on 3D positions of atoms in protein sites. The framework uses evolutionary relationships as self-supervision signals, enabling it to capture structure-function relationships. COLLAPSE demonstrated exceptional performance across various tasks, including PPIs and mutation stability prediction, outperforming standard benchmarks.

In terms of interaction prediction between human and virus proteins, Yang et al. [112] presented an innovative approach combining a Siamese CNN architecture with a multi-layer perceptron. The researchers introduced two transfer learning methods, termed ’frozen’ and ’fine-tuning’. These were used to predict interactions in a target human-virus domain, drawing from training in a source human-virus domain. Particularly, the ’frozen’ type transfer learning approach was applied to predict human-SARS-CoV-2 PPIs, uncovering interactions that are topologically and functionally similar to experimentally known interactions.

## 11. Other Emerging Topics for Protein–Protein Interactions

As the field of PPIs continues to grow, a variety of innovative and promising research topics are coming to the fore. These topics often revolve around novel applications of machine learning techniques or aim to address more specific and complex aspects of PPI prediction. This section provides an overview of some of these emerging topics in the field of PPIs, highlighting the broad scope and diversity of research that is currently being undertaken.

One of the key emerging areas involves the prediction of specific aspects of PPIs beyond merely identifying whether an interaction occurs. This includes predicting the binding sites of PPIs, understanding residue-residue interactions across protein interfaces, and determining protein–protein association rates. Each of these topics poses unique challenges and has the potential to contribute valuable insights into the mechanisms of PPIs.

Another noteworthy direction is the development of models that combine different types of features or use multiple learning techniques in a hybrid approach. These models often aim to take advantage of the strengths of different methods or to compensate for their individual weaknesses. For instance, some models may combine handcrafted and learned features, utilize both deep learning and gradient boosting methods, or integrate deep learning and reinforcement learning.

An additional trend in the field pertains to the application of deep learning methods to specific subsets of PPIs. Examples include the prediction of PPIs for specific organisms, such as plants, the study of interactions between humans and viruses, or the analysis of PPIs in specific subcellular locations, such as mitochondria. In each case, the uniqueness of the application necessitates the development of specialized models and approaches.

Furthermore, the rise of powerful deep learning methods, such as AlphaFold [26,29], is paving the way for novel applications and breakthroughs in the field of PPIs. The ability of these methods to predict protein structures with remarkable accuracy has implications for predicting PPIs, as well as for other related tasks, such as protein docking and protein complex modeling. It is anticipated that the use of these advanced models will become an increasingly prevalent topic in PPI research.

There is growing interest in utilizing deep learning techniques for the analysis of protein sequences and the extraction of valuable insights from these sequences. This encompasses a wide range of tasks, from predicting interaction sites in specific types of proteins, such as transmembrane proteins, to identifying coevolution patterns in protein families.

Recent studies have showcased diverse and innovative approaches for predicting PPIs using deep learning. These methods encompass various aspects, such as PPI binding site prediction, application of PPI analysis using existing tools, multi-label PPI prediction, protein docking decoys evaluation, protein interaction interface region prediction, protein complex modeling, and biomedical relation extraction. By leveraging protein sequence information, predicted structures, coevolution signals, joint multiple sequence alignments, and structural properties of proteins, these approaches have demonstrated remarkable performance improvements in PPI prediction accuracy and robustness. These advancements highlight the versatility and effectiveness of deep learning techniques in unraveling the complexities of PPIs and their implications in diverse biomedical research areas. Table 10 offers a summary of recent studies conducted.

Several studies have demonstrated the efficacy of various deep learning methods. For instance, Nikam et al. [128], developed DeepBSRPred, a deep-learning-based approach that predicts PPI binding sites using protein sequence information and predicted structures. Similarly, Tran et al. [137], introduced DeepCF-PPI, which combined handcrafted and learned features for PPI prediction, while Zhong et al. [146] proposed a multi-hop neural network model to predict multi-label PPIs.

In a similar vein, some researchers have focused on developing deep learning methods that leverage structural properties of proteins to improve PPI predictions. For instance, Han et al. [119] applied PointNet for protein docking decoys evaluation, enhancing ranking of near-native models. Furthermore, Dai and Bailey-Kellogg [116] presented PInet, a Geometric Deep Neural Network that predicts protein interaction interface regions from point clouds encoding the structures of two partner proteins.

Deep learning methods have also been applied to protein complex modelling and protein function prediction. Yin et al. [144] benchmarked the use of AlphaFold for protein complex modeling. Humphreys et al. [120] employed a similar strategy, using proteome-wide amino acid coevolution analysis and deep-learning-based structure modeling for systematic identification and building accurate models of core eukaryotic protein complexes. Additionally, Burke et al. [115] demonstrated the potential of AlphaFold2 in predicting structures for human protein interactions.

A few studies have concentrated on predicting PPIs based on coevolution signals from joint multiple sequence alignments. For instance, Pei et al. [131] employed AlphaFold to predict PPIs and their interfaces for proteins based on these signals. Similarly, Pei et al. [130] utilized deep learning methods RoseTTAFold and AlphaFold for analyzing coevolution of human proteins in mitochondria and modeling protein complexes.

Several novel approaches have also been proposed for predicting protein–protein interactions. These include GRNN-PPI by Xu et al. [141], TAGPPI by Song et al. [133], and DeepHomo2.0 by Lin et al. [124].

Lastly, several studies have been dedicated to the application of deep learning in biomedical relation extraction. Notably, Zhu et al. [147] proposed PACNN + RL, a hybrid deep learning and reinforcement learning method for this task. On the other hand, Jovine [121] used AlphaFold2 and ColabFold to investigate the activation and polymerization of uromodulin, thus showcasing the practical applicability of these methods in biomedicine.

## 12. Challenges and Future Directions in Recent Studies

Despite the remarkable advancements in employing deep learning models, particularly GNNs, GCNs, CNNs, autoencoders, and representation learning for PPI prediction, several challenges persist that need to be addressed. The ability of these models to predict PPIs often hinges on the availability and quality of training data, the integration of diverse data sources, model complexity, and interpretability. These challenges must be addressed to facilitate further improvements in PPI prediction and to understand biological systems at a more granular level.

One key challenge pertains to the availability and quality of PPI datasets. In several studies such as the work by Baranwal et al. [32] and Williams et al. [48], the high predictive performance of the models is reliant on robust, balanced datasets. Unfortunately, in the biological sciences, many datasets often contain imbalanced class distributions and noise, leading to biased model predictions and overfitting. Future research must therefore focus on developing strategies to cope with these issues, such as advanced data augmentation techniques, robust regularization methods, and ensemble modeling.

Moreover, while the integration of multimodal data sources and diverse biological features has shown promise in enhancing prediction performance, as evidenced by Albu et al. [30] and Kim et al. [37], it also poses challenges. Managing and harmonizing heterogeneous data types to prevent information loss, while ensuring efficient computation, remains a non-trivial task. Future studies need to explore better methods for feature extraction, selection, and fusion from various data sources to ensure an efficient and effective learning process.

The trade-off between model complexity and interpretability is another substantial challenge. As seen in studies like Soleymani et al. [77] and Chen et al. [53], deep learning models can be highly complex with numerous layers and nodes, leading to improved predictive performance. However, this complexity can often compromise interpretability, making it challenging to extract biological insights from the models. To address this, the development of techniques that enhance model transparency and interpretability is crucial. This may involve, for example, the use of attention mechanisms, saliency maps, and other explainable AI techniques.

One area requiring further exploration is the applicability of these models to emerging and interdisciplinary domains. Studies such as those conducted by Asim et al. [66] and Ray et al. [75] show the potential of these methods for viral–host PPI prediction and disease research, respectively. However, many other potential applications are yet to be explored in depth, such as the application of deep learning models for drug discovery, personalized medicine, and environmental genomics. Encouragingly, the progress made thus far provides a solid foundation for future research directions in these exciting areas.

Another recent and arguably crucial breakthrough in PPI prediction pertains to the prediction of structural information. One of the most significant and possibly the most challenging tasks in this regard is the prediction of the structure of protein–protein complexes. The application of deep learning has also extended to this challenge, with tools such as AlphaFold2 and its variants leading the charge [184].

AlphaFold and AlphaFold2 has shown remarkable levels of accuracy in modelling single chain protein structures [29,184]. This system can predict three-dimensional structures of proteins from amino acid sequences with atomic-level accuracy. In 2020, AlphaFold2 won the CASP14, and later it released structures of more than 200 million proteins, covering almost all known proteins on the planet [184].

Despite these achievements, accurately predicting the structures of protein–protein complexes remains a significant challenge. AlphaFold2 and its subsequent variants, despite being state-of-the-art predictors, still show room for improvement in this area. For instance, in a recent application of AlphaFold2 for the prediction of heterodimeric protein complexes, the tool generated models with acceptable quality for only 63% of the dimers [26]. While this is a promising result, it indicates that the problem of accurately predicting protein–protein complex structures is far from solved.

Given the ongoing challenges and limitations of current deep learning tools in predicting the structures of protein–protein complexes, several promising avenues for further research and development have emerged. One of these is the concept of “hot-spots”, regions of amino acid residues on the PPI interface that contribute significantly to binding-free energy. By focusing on these hot-spots, researchers may be able to design more effective PPI drugs, as small molecule drugs only need to target these regions to intervene in PPIs [25].

Another promising approach is the application of GNNs to predict PPIs. For instance, a study employed GCN and GAT to predict PPIs, utilizing protein structural information and sequence features. The protein’s amino acid network, also known as the residue contact network, was represented as a graph, where each node is a residue. This graph-based approach demonstrated superior performance over previous leading methods, suggesting that GNNs can be a powerful tool for PPI prediction [36].

The Fold-and-Dock approach has also shown potential for improving the prediction of PPIs. In this approach, two proteins are folded and docked simultaneously, which can provide more accurate results for predicting the structure of protein pairs. For instance, PconsDock, a fold-and-dock algorithm, has been developed to predict the structure of protein pairs where alternative methods fail [185]. However, this protocol still has limitations, as there remains a large set of protein–protein pairs where it fails. Future work is proposed to continue developing PconsDock by investigating improved methods to identify interaction protein sequences and developing improved deep learning methods to identify the contacts accurately.

These developments underline the importance of integrating various strategies and techniques to advance our ability to predict PPIs. The discovery of hot-spots, the application of GNNs, and the development of protocols represent promising directions for further advancements in the field of PPI prediction, which could significantly impact areas such as drug discovery and protein design.

While the use of deep learning models for PPI prediction has witnessed considerable progress, challenges remain that need to be addressed. By tackling these issues, the future of PPI prediction looks promising, with potential impacts not only on our understanding of biological systems but also on various practical applications such as drug discovery and disease diagnosis. With ongoing research and methodological enhancements, we can anticipate further advancements in this field.

## 13. Conclusions

In conclusion, the rapidly evolving landscape of deep learning presents a transformative platform for predicting PPIs. This synthesis of recent studies from 2021 to 2023 provides a pivotal compass in navigating the wealth of advancements that have unfolded within this highly dynamic field. As encapsulated within this review, the diversity and sophistication of deep learning techniques being applied to PPI prediction underscore this domain’s robust and innovative trajectory.

The myriad of deep learning methodologies, including GNNs, CNNs, Autoencoders, RNNs, Attention Mechanisms and Transformers, Multi-task and Multi-modal Learning, and Transfer Learning, each exhibits unique merits and characteristics in the context of PPI prediction. These powerful computational tools, endowed with the capability to distill intricate patterns within vast and complex datasets, continue to revolutionize our understanding of protein interactions and, by extension, biological systems at large.

This review serves as a testament to the potential of deep learning in not only facilitating the prediction of PPIs but also in unraveling the complexity inherent in their nature. It embodies a comprehensive resource for established researchers and newcomers in the field, equipping them with the necessary insights and references to propel their scientific endeavors. The fact that this domain continues to flourish at an unprecedented pace makes the timely amalgamation of these advancements within this review even more crucial.

In the rapidly advancing frontiers of computational biology, it is crucial to remain cognizant of emerging methodologies and their potential applications. This review, therefore, not only provides an up-to-date perspective on the current state-of-the-art but also underscores the importance of continuous learning and adaptation in this field. As we proceed forward, these deep learning methodologies are anticipated to continue to evolve, potentially reshaping our understanding and prediction of PPIs and ushering in novel strategies for biological inquiry and therapeutic development.

In the face of these evolving methodologies, it is the responsibility of the scientific community to scrutinize, validate, and contextualize these tools. Therefore, we hope this review will stimulate further discourse, innovation, and collaboration in applying deep learning techniques for PPI prediction and ultimately contribute to the acceleration of discoveries in this pivotal domain.

## Figures and Tables

**Table 1:** Overview of Deep Learning Methods for Protein–Protein Interactions.

Deep Learning Methods	Brief Description	Studies
Graph Neural Networks (GNNs)	Utilize graph data processing with deep learning	Albu et al. [30], Azadifar and Ahmadi [31], Baranwal et al. [32], Dai et al. [33], Gao et al. [34], Hinnerichs and Hoehndorf [35], Jha et al. [36], Kim et al. [37], Kishan et al. [38], Mahbub and Bayzid [39], Quadrini et al. [40], Reau et al. [41], Saxena et al. [42], Schapke et al. [43], St-Pierre Lemieux et al. [44], Strokach et al. [45], Wang et al. [46], Wang et al. [47], Williams et al. [48], Yuan et al. [49], Zaki et al. [50], Zhou et al. [51], Zhou et al. [52]
Convolutional Neural Networks (CNNs)	Utilize spatial data processing with deep learning	Chen et al. [53], Gao et al. [54], Guo et al. [55], Hu et al. [56], Hu et al. [57], Kozlovskii and Popov [58], Mallet et al. [59], Song et al. [60], Tsukiyama and Kurata [61], Wang et al. [62], Xu et al. [63], Yang et al. [64], Yuan et al. [65]
Representation Learning and Autoencoder	Utilize autoencoding for learning representations with deep learning	Asim et al. [66], Czibula et al. [67], Hasibi and Michoel [68], Ieremie et al. [69], Jha et al. [70], Jiang et al. [71], Liu et al. [72], Nourani et al. [73], Orasch et al. [74], Ray et al. [75], Sledzieski et al. [76], Soleymani et al. [77], Wang et al. [78], Yue et al. [79]
Recurrent Neural Networks (including LSTM)	Utilize sequential data processing with deep learning	Alakus and Turkoglu [80], Aybey and Gumus [81], Fang et al. [82], Li et al. [83], Mahdipour et al. [84], Ortiz-Vilchis et al. [85], Szymborski and Emad [86], Tsukiyama et al. [87], Zeng et al. [88], Zhang et al. [89], Zhou et al. [90]
Attention Methods and Transformers	Based on attention mechanism and position-specific encoding with deep learning	Asim et al. [91], Baek et al. [92], Li et al. [93], Li et al. [94], Nambiar et al. [95], Tang et al. [96], Warikoo et al. [97], Wu et al. [98], Zhang and Xu [99], Zhu et al. [100]
Multi-task and Multi-modal Learning	Perform multiple task or use multiple types of data simultaneously	Capel et al. [101], Li et al. [102], Linder et al. [103], Pan et al. [104], Peng et al. [105], Schulte-Sasse et al. [106], Thi Ngan Dong et al. [107], Zheng et al. [108]
Transfer Learning	Use pretrained deep learning models for feature extraction	Chen et al. [109], Derry and Altman [110], Si and Yan [111], Yang et al. [112], Zhang et al. [113]
Generic/Applications (including MLP) and Others	Includes models that do not fit specifically into other categories, or using PPIs as inputs of deep learning models	Abdollahi et al. [114], Burke et al. [115], Dai and Bailey-Kellogg [116], Dholaniya and Rizvi [117], Dhusia and Wu [118], Han et al. [119], Humphreys et al. [120], Jovine [121], Kang et al. [122], Li et al. [123], Lin et al. [124], Ma et al. [125], Madani et al. [126], Mahapatra et al. [127], Nikam et al. [128], Pan et al. [129], Pei et al. [130], Pei et al. [131], Singh et al. [132], Song et al. [133], Sreenivasan et al. [134], Stringer et al. [135], Sun and Frishman [136], Tran et al. [137], Wang et al. [138], Wee and Xia [139], Xie and Xu [140], Xu et al. [141], Yan and Huang [142], Yang et al. [143], Yin et al. [144], Zhang et al. [145], Zhong et al. [146], Zhu et al. [147], Zhu et al. [148]

**Table 2:** Journals of Publication.

Journal	Counts	Percentage (%)
Bioinformatics	21	17.6
Briefings in Bioinformatics	12	10.1
BMC Bioinformatics	12	10.1
IEEE-ACM Transactions on Computational Biology and Bioinformatics	7	5.9
Computational and Structural Biotechnology Journal	4	3.4
Frontiers in Genetics	4	3.4
Computers in Biology and Medicine	3	2.5
BMC Genomics	2	1.7
IEEE Access	2	1.7
IEEE Journal of Biomedical and Health Informatics	2	1.7
Scientific Reports	2	1.7
Science	2	1.7
Protein Science	2	1.7
Journal of Proteome Research	2	1.7
Interdisciplinary Sciences-Computational Life Sciences	2	1.7
Mathematics	2	1.7
Journal of Chemical Information and Modeling	2	1.7
Nature Machine Intelligence	2	1.7
Others (<2 Publication)	34	28.6

**Table 3:** Summary of Contributions in Studies on Graph Neural Networks for Protein–Protein Interactions. Note that each study employed varied datasets, cross-validation methods, and simulation settings for evaluation, making direct comparisons potentially inconclusive. The highest reported accuracy is presented when models were assessed using multiple datasets.

Author	Metrics and Results	Contributions
Albu et al. [30]	AUC: 0.92 AUPRC: 0.93	Developed MM-StackEns, a deep multimodal stacked generalization approach for predicting PPIs.
Azadifar and Ahmadi [31]	AUC: 0.8847	Introduced a semi-supervised learning method for prioritizing candidate disease genes.
Baranwal et al. [32]	ACC: 0.9889 MCC: 0.9779 AUC: 0.9955	Presented Struct2Graph, a GAT designed for structure-based predictions of PPIs.
Dai et al. [33]	MSE: 0.2446 PCC: 0.8640	Formulated a method for predicting protein abundance from scRNA-seq data.
Gao et al. [34]	ACC: 0.778	Developed the Substructure Assembling Graph Attention Network (SA-GAT) for graph classification tasks.
Hinnerichs and Hoehndorf [35]	AUC: 0.94	Devised DTI-Voodoo, a method combining molecular features and PPI networks to predict drug-target interactions.
Jha et al. [36]	ACC: 0.9813 MCC: 0.9520 AUC: 0.9828 AUPRC: 0.9886	Proposed the use of GCN and GAT to predict PPIs.
Kim et al. [37]	Precision: 0.60 F1: 0.52 NMI: 0.404	Proposed DrugGCN, a GCN for drug response prediction using gene expression data.
Kishan et al. [38]	AUC: 0.936 AUPRC: 0.941	Developed a higher-order GCN for biomedical interaction prediction.
Mahbub and Bayzid [39]	ACC: 0.715 MCC: 0.27 AUC: 0.719 AUPRC: 0.405	Introduced EGRET, an edge aggregated GAT for PPI site prediction.
Quadrini et al. [40]	ACC: 0.731 MCC: 0.054 AUC: 0.588	Explored hierarchical representations of protein structure for PPI site prediction.
Reau et al. [41]	AUC: 0.85	Developed DeepRank-GNN, a graph neural network framework for learning interaction patterns.
Saxena et al. [42]	ACC: 0.9113 F1: 0.90	Proposed a network centrality based approach combined with GCNs for link prediction.
Schapke et al. [43]	AUC: 0.9043 AUPRC: 0.7668	Developed EPGAT, an essentiality prediction model based on GATs.
St-Pierre Lemieux et al. [44]	ACC: 0.84 MCC: 0.94	Presented several geometric deep-learning-based approaches for PPI predictions.
Strokach et al. [45]	Spearman’s *R*: 0.62	Described ELASPIC2 (EL2), a machine learning model for predicting mutation effects on protein folding and PPI.
Wang et al. [46]	ACC 0.9365 MCC 0.4301 AUC 0.6068	Developed SIPGCN, a deep learning model for predicting self-interacting proteins.
Wang et al. [47]	ACC: 0.413	Introduced PLA-GNN, a method for identifying alterations of protein subcellular locations.
Williams et al. [48]	AUC: 0.85	Developed DockNet, a protein–protein interface contact prediction model.
Yuan et al. [49]	ACC: 0.776 MCC: 0.333 AUC: 0.786 AUPRC: 0.429	Proposed GraphPPIS, a deep graph-based framework for PPI site prediction.
Zaki et al. [50]	F1: 0.616	Developed a method for detecting protein complexes in PPI data using GCNs.
Zhou et al. [51]	AUC: 0.5916 AP: 0.85	Conducted a comparative study on various graph neural networks for PPI prediction.
Zhou et al. [52]	ACC: 0.856 F1: 0.569 AUC: 0.867 AUPRC: 0.574	Presented AGAT-PPIS, an augmented graph attention network for PPI site prediction.

**Table 4:** Summary of Contributions in Studies on Convolutional Neural Networks for Protein–Protein Interactions. Note that each study employed varied datasets, cross-validation methods, and simulation settings for evaluation, making direct comparisons potentially inconclusive. The highest reported accuracy is presented when models were assessed using multiple datasets.

Author	Metrics and Results	Contributions
Chen et al. [53]	ACC: 0.9303 F1: 0.9268 MCC: 0.8609	Developed DCSE, a sequence-based model using MCN and MBC for feature extraction and PPI prediction.
Gao et al. [54]	ACC: 0.9534 MCC: 0.9086 AUC: 0.9824	Introduced EResCNN, an ensemble residual CNN integrating diverse feature representations for PPI prediction.
Guo et al. [55]	ACC: 0.884 PCC: 0.366	Introduced TRScore, a 3D RepVGG-based scoring method for ranking protein docking models.
Hu et al. [56]	ACC: 0.9755 MCC: 0.9515 F1: 0.9752	Developed DeepTrio, a PPI prediction tool using mask multiple parallel convolutional neural networks.
Hu et al. [57]	ACC: 0.859 MCC: 0.399 AUC: 0.824 AUPRC: 0479	Developed D-PPIsite, a deep residual network integrating four sequence-driven features for PPI site prediction.
Kozlovskii and Popov [58]	AUC: 0.91 MCC: 0.49	Developed BiteNet, a 3D convolutional neural network method for protein–peptide binding site detection.
Mallet et al. [59]	ACC≃ 0.70	Developed InDeep, a 3D fully convolutional network tool for predicting functional binding sites within proteins.
Song et al. [60]	ACC: 0.776 MCC: 0.333 AUC: 0.786 AUPRC: 0.429	Presented a method for clustering spatially resolved gene expression using a graph-regularized convolutional neural network, leveraging the PPI network graph.
Tsukiyama and Kurata [61]	ACC: 0.956 F1: 0.955 MCC: 0.912 AUC: 0.988	Proposed Cross-attention PHV, a neural network utilizing cross-attention mechanisms and 1D-CNN for human-virus PPI prediction.
Wang et al. [62]	ACC: 0.784 MCC:0.5685	Proposed an enhancement to a 2D CNN using Sequence-Statistics-Content (SSC) protein sequence encoding format for PPI tasks.
Xu et al. [63]	ACC: 0.9617 F1: 0.9257	Introduced OR-RCNN, a PPI prediction framework based on ordinal regression and recurrent convolutional neural networks.
Yang et al. [64]	AUC: 0.885 MCC: 0.390	Proposed PhosIDN, an integrated deep neural network combining sequence and PPI information for improved prediction of protein phosphorylation sites.
Yuan et al. [65]	ACC: 0.9680	Presented a deep-learning-based approach combining a semi-supervised SVM classifier and a CNN for constructing complete PPI networks.

**Table 5:** Summary of Contributions in Studies on Representation Learning for Protein–Protein Interactions. Note that each study employed varied datasets, cross-validation methods, and simulation settings for evaluation, making direct comparisons potentially inconclusive. The highest reported accuracy is presented when models were assessed using multiple datasets.

Author	Metrics and Results	Contributions
Asim et al. [66]	ACC: 0.82 MCC: 0.6399 F1: 0.6399 AUC: 0.88	Developed LGCA-VHPPI, a deep forest model for effective viral-host PPI prediction using statistical protein sequence representations.
Czibula et al. [67]	ACC: 0.983 F1: 0.984 AUC: 0.985	Introduced AutoPPI, an ensemble of autoencoders designed for PPI prediction, yielding strong performance on several datasets.
Hasibi and Michoel [68]	MSE: 0.133	Demonstrated a Graph Feature Auto-Encoder that utilizes the structure of gene networks for effective prediction of node features.
Ieremie et al. [69]	AUC: 0.939	Proposed TransformerGO, a model predicting PPIs by modeling the attention between sets of Gene Ontology (GO) terms.
Jha et al. [70]	ACC: 0.8355 F1: 0.8349	Utilized a stacked auto-encoder for PPI prediction, showcasing effective feature extraction approach for addressing PPI problems.
Jiang et al. [71]	ACC: 0.990 MCC: 0.975 F1 0.990	Introduced DHL-PPI, a deep hash learning model to predict all-against-all PPI relationships with reduced time complexity.
Liu et al. [72]	AUC: 0.658	Designed GraphPheno, a graph autoencoder-based method to predict relationships between human proteins and abnormal phenotypes.
Nourani et al. [73]	AP: 0.7704	Presented TripletProt, a deep representation learning approach for proteins, proving effective for protein functional annotation tasks.
Orasch et al. [74]	AUC 0.88	Presented a new deep learning architecture for predicting interaction sites and interactions of proteins, showing state-of-the-art performance.
Ray et al. [75]	ND	Presented a deep learning methodology for predicting high-confidence interactions between SARS-CoV2 and human host proteins.
Sledzieski et al. [76]	AUPRC: 0.798	Presented D-SCRIPT, a deep-learning model predicting PPIs using only protein sequences, maintaining high accuracy across species.
Soleymani et al. [77]	ACC: 0.9568 AUC: 0.9600	Proposed ProtInteract, a deep learning framework for efficient prediction of protein–protein interactions.
Wang et al. [78]	ACC: 0.633 AUC: 0.681 AUPRC: 0.339	Introduced DeepPPISP-XGB, a method integrating deep learning and XGBoost for effective prediction of PPI sites.
Yue et al. [79]	ACC: 0.9048 AUC: 0.93	Proposed a deep learning framework to identify essential proteins integrating features from the PPI network, subcellular localization, and gene expression profiles.

**Table 6:** Summary of Contributions in Studies on Recurrent Neural Networks for Protein-Protein Interactions. Note that each study employed varied datasets, cross-validation methods, and simulation settings for evaluation, making direct comparisons potentially inconclusive. The highest reported accuracy is presented when models were assessed using multiple datasets.

Author	Metrics and Results	Contributions
Alakus and Turkoglu [80]	ACC: 0.9776 F1: 0.7942 AUC: 0.89	Proposed a deep learning method for predicting protein interactions in SARS-CoV-2.
Aybey and Gumus [81]	AUC: 0.715 MCC: 0.227 F1: 0.330	Developed SENSDeep, an ensemble deep learning method, for predicting protein interaction sites.
Fang et al. [82]	ACC: 0.9445 ROC: 0.94	Employed an integrated LSTM-based approach for predicting protein–protein interactions in plant-pathogen studies.
Li et al. [83]	ACC: 0.848 AUC: 0.746 AUPRC: 0.326	Proposed DELPHI, a deep learning suite for PPI-binding sites prediction.
Mahdipour et al. [84]	ACC: 1.0 F1: 1.0	Introduced RENA, an innovative method for PPI network alignment using a deep learning model.
Ortiz-Vilchis et al. [85]	ACC: 0.949	Utilized LSTM model to generate relevant protein sequences for protein interaction prediction.
Szymborski and Emad [86]	AUC: 0.978 AUPRC: 0.974	Introduced RAPPPID, an AWD-LSTM twin network, to predict protein–protein interactions.
Tsukiyama et al. [87]	ACC: 0.985 AUC: 0.976	Presented LSTM-PHV, a model for predicting human-virus protein–protein interactions.
Zeng et al. [88]	ACC: 0.9048 F1: 0.7585	Introduced a deep learning framework for identifying essential proteins by integrating multiple types of biological information.
Zhang et al. [89]	ACC: 0.83 AUC: 0.93	Presented protein2vec, an LSTM-based approach for predicting protein–protein interactions.
Zhou et al. [90]	ACC: 0.75	Implemented LSTM-based model for predicting protein–protein interaction residues using frustration indices.

**Table 7:** Summary of Contributions in Studies on Attention and Transformer for Protein-Protein Interactions. Note that each study employed varied datasets, cross-validation methods, and simulation settings for evaluation, making direct comparisons potentially inconclusive. The highest reported accuracy is presented when models were assessed using multiple datasets.

Author	Metrics and Results	Contributions
Asim et al. [91]	ACC: 0.926 F1: 0.9195 MCC: 0.855	Proposed ADH-PPI, an attention-based hybrid model with superior accuracy for PPI prediction.
Baek et al. [92]	ACC: 0.868 MCC: 0.768 F1: 0.893 AUC: 0.982	Utilized a three-track neural network integrating information at various dimensions for protein structure and interaction prediction.
Li et al. [93]	F1: 0.925	Offered a PPI relationship extraction method through multigranularity semantic fusion, achieving high F1-scores.
Li et al. [94]	ACC: 0.9519 MCC: 0.9045 AUC: 0.9860	Introduced SDNN-PPI, a self-attention-based PPI prediction method, achieving up to 100% accuracy on independent datasets.
Nambiar et al. [95]	ACC: 0.98 AUC: 0.991	Developed a Transformer neural network that excelled in protein interaction prediction and family classification.
Tang et al. [96]	ACC: 0.631 F1: 0.393	Proposed HANPPIS, an effective hierarchical attention network structure for predicting PPI sites.
Warikoo et al. [97]	F1: 0.86	Introduced LBERT, a lexically aware transformer-based model that outperformed state-of-the-art models in PPI tasks.
Wu et al. [98]	AUPRC: 0.8989	Presented CFAGO, an efficient protein function prediction model integrating PPI networks and protein biological attributes.
Zhang and Xu [99]	ACC: 0.856	Introduced a kernel ensemble attention method for graph learning applied to PPIs, showing competitive performance.
Zhu et al. [100]	ACC: 0.934 F1: 0.932 AUC: 0.935	Introduced the SGAD model, improving the performance of Protein Interaction Network Reconstruction.

**Table 8:** Summary of Contributions in Studies on Multi-task or Multi-modal Models for Protein-Protein Interactions. Note that each study employed varied datasets, cross-validation methods, and simulation settings for evaluation, making direct comparisons potentially inconclusive. The highest reported accuracy is presented when models were assessed using multiple datasets.

Author	Metrics and Results	Contributions
Capel et al. [101]	AUC: 0.7632 AUPRC: 0.3844	Proposed a multi-task deep learning approach for predicting residues in PPI interfaces.
Li et al. [102]	AUC: 0.895 AUPRC: 0.899	Developed EP-EDL, an ensemble deep learning model for accurate prediction of human essential proteins.
Linder et al. [103]	AUC: 0.96	Introduced scrambler networks to improve the interpretability of neural networks for biological sequences.
Pan et al. [104]	ACC: 0.8947 MCC: 0.7902 AUC: 0.9548	Proposed DWPPI, a network embedding-based approach for PPI prediction in plants.
Peng et al. [105]	AUC: 0.9116 AUPRC: 0.8332	Introduced MTGCN, a multi-task learning method for identifying cancer driver genes.
Schulte-Sasse et al. [106]	AUPRC: 0.76	Developed EMOGI, integrating *MULTIOMICS* data with PPI networks for cancer gene prediction.
Thi Ngan Dong et al. [107]	AUC: 0.9804 F1: 0.9379	Developed a multitask transfer learning approach for predicting virus-human and bacteria-human PPIs.
Zheng et al. [108]	AUPRC: 0.965	Developed DeepAraPPI, a deep learning framework for predicting PPIs in Arabidopsis thaliana.

**Table 9:** Summary of Contributions in Studies on Transfer Learning for Protein–Protein Interactions. Note that each study employed varied datasets, cross-validation methods, and simulation settings for evaluation, making direct comparisons potentially inconclusive. The highest reported accuracy is presented when models were assessed using multiple datasets.

Author	Metrics and Results	Contributions
Chen et al. [109]	ACC: 0.9745	Developed TNNM, a model for predicting essential proteins with superior performance on two public databases.
Derry and Altman [110]	AUC: 0.881	Proposed COLLAPSE, a framework for identifying protein structural sites, demonstrating excellent performance in various tasks including PPIs.
Si and Yan [111]	AvgPR: 0.576	Presented DRN-1D2D_Inter, a deep learning method for inter-protein contact prediction with enriched input features.
Yang et al. [112]	ACC: 0.9865 F1: 0.9236 AUPRC: 0.974	Utilized a Siamese CNN and a multi-layer perceptron for human-virus PPI prediction, applying transfer learning for human-SARS-CoV-2 PPIs.
Zhang et al. [113]	AvgPR: 0.6596	Introduced HDIContact, a deep learning framework for inter-protein residue contact prediction, showcasing promising results for understanding PPI mechanisms.

**Table 10:** Summary of Contributions in Other Emerging Topics for Protein–Protein Interactions.

Author	Contributions
Abdollahi et al. [114]	Developed WinBinVec, a window-based deep learning model to identify cancer PPIs.
Burke et al. [115]	Demonstrated a potential of AlphaFold2 in predicting structures for protein interactions.
Dai and Bailey-Kellogg [116]	Presented PInet, a Geometric Deep Neural Network that predicts PPI from point clouds encoding the structures of two partner proteins.
Dholaniya and Rizvi [117]	Examined the efficacy of various sequence-based descriptors in predicting PPIs.
Dhusia and Wu [118]	Proposed a neural network model to estimate protein–protein association rates.
Han et al. [119]	Applied PointNet for protein docking decoys evaluation.
Humphreys et al. [120]	Used proteome-wide amino acid coevolution analysis and deep-learning-based structure modeling for core eukaryotic protein complexes.
Jovine [121]	Used AlphaFold2 and ColabFold to investigate the activation of uromodulin.
Kang et al. [122]	Introduced HN-PPISP, a hybrid neural network for PPI site prediction.
Li et al. [123]	Proposed HDOCKsite, an approach incorporating interface residue restraints into protein–protein docking.
Lin et al. [124]	Proposed DeepHomo2.0, a model that predicts PPIs of homodimeric complexes.
Ma et al. [125]	Proposed MSF-DTA, a deep-learning-based method using PPI information for predicting drug-target affinity.
Madani et al. [126]	Proposed CGAN-Cmap, a novel hybrid model for protein contact map prediction.
Mahapatra et al. [127]	Developed DNN-XGB, a hybrid classifier for PPI prediction combining DNN and XGBoost.
Nikam et al. [128]	Developed DeepBSRPred for predicting PPI binding sites using protein sequence.
Pan et al. [129]	Presented a framework combining discrete Hilbert transform (DHT) with DNN for plant PPI prediction.
Pei et al. [130]	Utilized deep learning methods for analyzing coevolution of human proteins in mitochondria and modeling protein complexes.
Pei et al. [131]	Employed AlphaFold to predict PPIs and interfaces for coevolution signals.
Singh et al. [132]	Introduced Topsy-Turvy, a sequence-based multi-scale model for PPI prediction.
Song et al. [133]	Proposed TAGPPI, an end-to-end framework to predict PPIs using protein sequences and graph learning method.
Sreenivasan et al. [134]	Developed MolPMoFiT for predicting protein clusters based on chemical structure.
Stringer et al. [135]	Developed PIPENN, an ensemble of neural networks for protein interface prediction from protein sequences.
Sun and Frishman [136]	Developed DeepTMInter, a novel approach for sequence-based prediction of interaction sites in alpha-helical transmembrane proteins.
Tran et al. [137]	Introduced DeepCF-PPI, combining handcrafted and learned features for PPI prediction.
Wang et al. [138]	Developed DeepViral, a deep learning method that predicts PPIs between humans and viruses using protein sequences and infectious disease phenotypes.
Wee and Xia [139]	Proposed PerSpect-EL, an ensemble learning model for protein–protein binding prediction.
Xie and Xu [140]	Developed GLINTER, a deep learning method for inter-protein contact prediction, using protein tertiary structures and a pretrained language model.
Xu et al. [141]	Developed GRNN-PPI, a PPI prediction algorithm for multiple datasets.
Yan and Huang [142]	Proposed DeepHomo, a deep learning model for predicting inter-protein residue-residue contacts across homo-oligomeric protein interfaces.
Yang et al. [143]	Examined interface and surface areas in protein–protein binding prediction.
Yin et al. [144]	Benchmarked the use of AlphaFold for protein complex modeling.
Zhang et al. [145]	Predicted functions and a PPI network for proteins in the minimal genome JCVI-syn3A.
Zhong et al. [146]	Presented a multi-hop neural network model for predicting multi-label PPIs.
Zhu et al. [147]	Proposed PACNN+RL, a hybrid deep learning and reinforcement learning method, for biomedical relation extraction.
Zhu et al. [148]	Introduced PPICT, a deep neural network designed to predict PTM inter-protein cross-talk.

## Data Availability

No new data were created or analyzed in this study.
